# Cannot Extubate a Newborn Patient after an Arterial Switch Operation? Check Major Aortopulmonary Collaterals!

**DOI:** 10.21470/1678-9741-2019-0109

**Published:** 2020

**Authors:** Ibrahim Cansaran Tanıdır, Erkut Ozturk, Murat Sahin, Sertac Haydin, Alper Guzeltas

**Affiliations:** 1 Department of Pediatric Cardiology, Saglik Bilimleri University, Istanbul Mehmet Akif Ersoy Thoracic and Cardiovascular Surgery Education and Research Hospital, Istanbul, Turkey.; 2 Department of Pediatric Cardiovascular Surgery, Saglik Bilimleri University, Istanbul Mehmet Akif Ersoy Thoracic and Cardiovascular Surgery Education and Research Hospital, Istanbul, Turkey.

**Keywords:** Transposition of the Great Vessels, Arterial Switch Operation, Airway Extubation, Cardiac Catheterization, Hemodynamics, Infants, Newborn, Humans

## Abstract

The standard treatment of transposition of the great arteries is the arterial switch operation (ASO). Despite successful surgical correction, patients cannot tolerate extubation after the operation. Major aortopulmonary collaterals (MAPCAs) are one of the rare causes of prolonged mechanical ventilation due to significant hemodynamic effects. We report a 28-day-old newborn with transposition of the great arteries and a ventricular septal defect (VSD) who underwent ASO and VSD closure. After postoperative extubation failed twice, four large MAPCAs were revealed during heart catheterization. After transcatheter closure of these four MAPCAs, the patient was extubated and discharged 27 days after the procedure.

**Table:** 

Abbreviations, acronyms & symbols	
ASO	= Arterial switch operation
LV	= Left ventricular
MAPCA	= Major aortopulmonary collateral
PA	= Pulmonary atresia
PDA	= Patent ductus arteriosus
TGA	= Transposition of the great arteries
ToF	= Tetralogy of Fallot
VSD	= Ventricular septal defect

## INTRODUCTION

Transposition of the great arteries (TGA) is the most common cyanotic congenital heart disease in the neonatal period. The arterial switch operation (ASO) has become the standard treatment of choice for TGA in the first few weeks of life^[1]^. Surgical complications in patients who underwent ASO, the presence of residual defects, myocardial dysfunction, the presence of pulmonary parenchyma, and comorbid problems may complicate extubation, thereby increasing the length of stay on a mechanical ventilator^[1,2]^.

Major aortopulmonary collateral (MAPCA) vessels represent rare abnormalities in association with TGA, and the possible symptoms of hemodynamic relevant MAPCAs are pulmonary volume overload, respiratory failure, and left atrial and ventricular dilatation, as well as dysfunction, failure to thrive, tachycardia, or arrhythmias^[3,4]^.

We report a 28-day-old newborn with TGA and ventricular septal defect (VSD). The patient underwent an ASO and a VSD closure. Unfortunately, he could not be extubated. Cardiac catheterization revealed MAPCAs, and the patient was extubated and discharged after successful transcatheter MAPCA embolization.

## CASE REPORT

The neonate was born at 40 weeks of gestation, and he weighed 3200 g. On postnatal 20^th^ day, he was referred to our clinic due to cyanosis. His physical examination was normal, except for central cyanosis (84% with pulse oximetry). Normal sinus rhythm with right axis and right ventricular dominance was present in the electrocardiography evaluation.

Echocardiography confirmed TGA (anterior to posterior relationship of the great vessels) with the usual branching pattern of the coronary arteries, a large perimembranous VSD (9 mm), a restrictive patent foramen ovale, and a 3 mm ductus arteriosus. There was no evidence of coarctation of the aorta. There was no left ventricular (LV) systolic dysfunction or outflow obstruction. Severe mitral insufficiency was present.

**Fig. 1:**
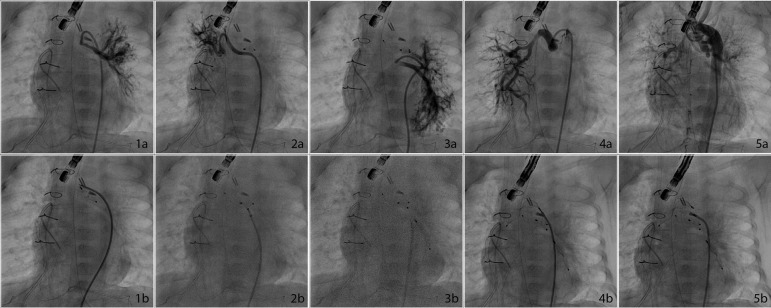
1a)Anteroposterior view of MAPCA feeding left sided pulmonary fields; 1b) After AVP-4 deployment; 2a) Anteroposterior view of MAPCA feeding right sided pulmonary fields; 2b) After ADOII-AS deployment; 3a) Anteroposterior view of MAPCA feeding left sided pulmonary fields; 3b) After AVP-4 deployment; 4a) Anteroposterior view of MAPCA feeding right sided pulmonary fields; 4b) After AVP-4 deployment; 5a) Aortogram before the procedure showing all MAPCAs; 5b) Anteroposterior view after the procedure. MAPCA=major aortopulmonary collateral

The patient successfully underwent an ASO and a VSD closure operation at 28 days of life. During surgery, there was no evidence of increased pulmonary venous return, and no unusual findings were encountered. Despite a seamless postoperative course, the patient was re-intubated after two extubation attempts. Echocardiography did not reveal any unusual condition other than the major mitral regurgitation, which was initially present. Seven days after the operation, the patient’s clinical condition worsened due to development of a lung infection, so antibiotic and inotropic support had to be started. Inotropic support was administered as milrinone (0.5 µg/kg/min) and a low dose of epinephrine (0.05 µg/kg/min). Diuretic treatment (furosemide and spironolactone-hydrochlorothiazide) was initiated on the decrease of urine output. However, in addition to adequate medical therapy, peritoneal dialysis was started due to anuria. Since a serial echocardiographic examination with color Doppler revealed enlargement of the left heart cavities and suspicious left-right transitions in the basal segment of the interventricular septum, we decided to perform a cardiac catheterization and angiography at the 11^th^ postoperative day. The coronary artery pattern was normal on angiography, and there was no residual VSD. On the other hand, multiple MAPCAs were observed on the aortic angiogram (Figure 1, 1A to 5A). Three MAPCAs were occluded with the Amplatzer Vascular Plug-4 (5 mm, 7 mm, and 8 mm) (St. Jude Medical Inc., St. Paul, Minnesota, USA) and one with the Amplatzer Duct Occluder Type II Additional Size™ (ADO-IIAS, St. Jude Medical Inc., St. Paul, Minnesota, USA) (Figure 1).

After the MAPCAs closure, mitral valve insufficiency decreased from significant to moderate degree. Extubation was achieved four days after MAPCA embolization. The patient was discharged on the 27^th^ postoperative day without any sequel. During a midterm follow-up, serial clinical and echocardiographic evaluations confirmed good clinical cardiac function and complete relief of the cardiac volume overload.

## DISCUSSION

TGA is one of the most common cyanotic heart diseases in neonates, 1 of 5000 live births. The incidence of aortic arch anomalies is 10% and may include, in order of frequency: coarctation, arch interruption, right aortic arch, and double aortic arch. The presence of enlarged bronchial collateral vessels has been widely documented in TGA^[3]^.

In the first weeks of gestation, together with the onset of the normal pulmonary arterial system formation, systemic-to-pulmonary collateral arteries normally start to regress as they are not preprogrammed to “survive” after birth. When there is an early underdevelopment of the pulmonary arterial system or the pulmonary valve, MAPCAs may persist, like in pulmonary atresia (PA) or Tetralogy of Fallot (ToF), thereby ensuring enough postnatal pulmonary blood flow. These heart defects are characterized by reduced oxygen saturation in blood postnatally (reduced pulmonary blood flow with right-to-left shunting via VSD and/or atrial septal defect in PA and ToF and parallel circulations with reduced mixing of blood in TGA)^[4,5]^.

Many of these aortopulmonary collateral arteries are clinically silent. A few cases have been described early after neonatal ASOs caused symptomatic cardiac overload or failure to wean from mechanical ventilation. Unfortunately, these anomalous vessels are rarely imaged before surgical repair, even by selective aortic angiography. It might be presumably due to vasoconstriction caused by local pulmonary hypoxemia as well as ductal ‘‘steal’’ that obscures the flow through them. Their presence might be suspected during surgical correction when significant left atrial venous return is noticed in the operative field during rewarming on cardiopulmonary bypass, although persistent vasoconstriction during surgery might make these arteries hemodynamically invisible, as was the case in our patient. Conversely, these vessels are easily detected on routine selective angiography after corrective surgery, possibly due to vasoconstriction relief^[4-7]^.

Although in most cases these vessels are very small and remain asymptomatic, sometimes they might be large enough to result in massive airway bleeding, systemic hypoxemia, pulmonary overperfusion, or combined LV and respiratory failure even early after corrective surgery^[8]^. This kind of “hemodynamically relevant MAPCA” incidence was found to be as high as 15% in cases with a diagnosis of simple TGA because of an early complicated postoperative course or difficult coronary transfer due to special coronary anatomy which underwent cardiac catheterization after an ASO^[4]^.

In the Wibf study^[4]^, they concluded that the echocardiography sensitivity was 53%, while a positive echo finding (flow in the color Doppler suspicious for a collateral vessel, left ventricle dysfunction, or backflow in descending aorta, left atrial/left ventricle dilatation) correlates well with a positive result in angiography (specificity 100%). Similarly, Maddali et al.^[5]^ found MAPCAs in angiography without any echocardiographic findings in their cases. In our case, we did not see any obvious MAPCA finding in echocardiography, but we were able to diagnose it with angiography.

It is recommended to close MAPCAs that are hemodynamically significant. Santoro et al.^[6]^ described the first successful coil embolization of multiple MAPCAs in a neonate after a successful ASO that resulted in the child’s rapid recovery. The largest series of 98 cases, published by Wibf et al.^[4]^, issued the largest analysis of TGA with MAPCAs. Overall, MAPCAs were detected in 20 of 98 (20%) TGA patients who underwent surgery and 20 of 37 (54%) patients who underwent cardiac catheterization due to an indication after surgery, and 15 of 37 (44%) MAPCAs needed to be embolized due to their hemodynamically significant side effects. Cardiologists prefer coil embolization of MAPCAs in their series. In this present case report, we closed multiple MAPCAs with AVP-4 and ADO-IIAS devices.

When we retrospectively analyze the patient, there are some overlooked points that must be discussed. Preoperative (also early postoperative) severe mitral regurgitation suggests that there could be excessive volume overload. First, we should consider the etiology of mitral regurgitation before the procedure and have a plan for that, either surgical repair or follow-up. Second, in a patient like this, it is quite unusual to see severe mitral regurgitation just as with VSD and patent ductus arteriosus (PDA). At this time, cardiac catheterization and angiogram or at least additional computed tomography scanning should have been performed to reveal hidden findings undetected by echo. On the other hand, these MAPCAs seem smaller when the PDA is still open, and after surgeons close the PDA, they may become larger. This may be a problem if we search for MAPCAs before the procedure and underestimate them.

## CONCLUSION

In conclusion, in patients who needed prolonged mechanical ventilation and were unable to wean from mechanical ventilation support after ASOs and patients with severe mitral regurgitation with volume overloading findings, MAPCAs should be investigated. Hemodynamically significant MAPCAs should be closed.

**Table:** 

Author's roles & responsibilities	
ICT	Design of the work; or the acquisition, analysis, or interpretation of data for the work; drafting the work or revising it critically for important intellectual content; final approval of the version to be published
EO	Design of the work; or the acquisition, analysis, or interpretation of data for the work; drafting the work or revising it critically for important intellectual content; final approval of the version to be published
MS	Design of the work; or the acquisition, analysis, or interpretation of data for the work; final approval of the version to be published
SH	Drafting the work or revising it critically for important intellectual content; final approval of the version to be published
AG	Drafting the work or revising it critically for important intellectual content; final approval of the version to be published
